# Synthesis and characterization of Ag-doped ZnO nanostructures and an assessment on their enhanced bacterial inhibition and photocatalytic degradation

**DOI:** 10.1039/d5ra09660j

**Published:** 2026-03-24

**Authors:** Vishwajeet. Aherkar, Ali Abdulmawjood Mohammed, Anmar A. Al-shimary, Rekha M. Ovhal, Shivaji Pawar, Ganesh Kamble, Panchsheela A. Ubale

**Affiliations:** a Department of Chemistry, Walchand College of Arts and Science Solapur Maharashtra 413001 India; b Department of Chemistry, Sangameshwar College Solapur Maharashtra-413001 India; c College of Science, Mustansiriyah University Baghdad-10001 Iraq; d Centre for Research and Technology Developments (CRTD), N.B. Navale Sinhgad College of Engineering Solapur 413255 Maharashtra India; e Department of Chemistry, Kolhapur Institute of Technology, College of Engineering (Empowered Autonomous) Kolhapur-41623 Maharashtra India; f N.K. Orchid College of Engineering and Technology Solapur-413002 Maharashtra India panchsheela_ubale@rediffmail.com +91-9370350152

## Abstract

In this study, zinc oxide (ZnO) and silver-doped ZnO nanoparticles with varying silver concentrations (1, 3, 5, and 7 mol%) were synthesized using a simple and cost-effective sol–gel method. The prepared nanoparticles were characterized in terms of their optical, structural, and morphological properties using ultraviolet-visible (UV-vis) spectroscopy, X-ray diffraction (XRD), field-emission scanning electron microscopy (FESEM), and high-resolution transmission electron microscopy (HRTEM). The study revealed that band gap decreases and particle size increases with increasing Ag content. The hexagonal wurtzite crystalline structure of ZnO and Ag-doped ZnO is confirmed by XRD. The photocatalytic activity of the synthesized nanoparticles toward methylene blue degradation, along with their antibacterial activity against *Staphylococcus aureus* and *Pseudomonas aeruginosa*, and antifungal activity against *Candida albicans*, was investigated.

## Abstract 2

In this study, we have synthesized zinc oxide (ZnO) and silver-doped zinc oxide nanoparticles with different molar concentrations of silver (1, 3, 5 and 7 mol%) using a simple and cost-effective sol–gel method.

## Graphical abstract

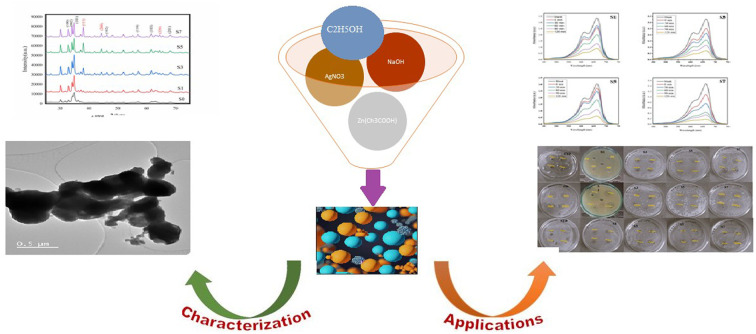

## 1. Introduction

The area of nanotechnology has shown great promise and has the potential to completely transform a number of sectors, including electronics, healthcare, and energy. Zinc oxide nanoparticles' performance has been found to be greatly improved by doping, which is the act of adding impurities to a semiconductor to change its electrical or optical properties.^1,2^ The incorporation of dopants like gallium, indium, and aluminium into the zinc oxide lattice has gained considerable scientific interest owing to their ability to improve multiple properties.^3^ Changes in zinc oxide nanoparticles' bandgap, optical properties, and electrical conductivity can have a significant impact on how well they perform in a variety of applications, including solar energy conversion, gas sensing, and photocatalysis.^4^ Dopants can change the surface energy and reactivity of zinc oxide nanoparticles, which can affect how these nanoparticles interact with certain molecules during gas sensing and catalysis applications.^5^ Furthermore, doping can modify the reactivity and surface chemistry of zinc oxide nanoparticles, thereby influencing their interaction with target molecules in sensing and catalytic applications.^6^ By gaining a deeper understanding of these effects, we can explore the potential of zinc oxide nanoparticles in other technological domains.

To enhance the properties of ZnO nanoparticles for applications in photocatalysis, sensing, optoelectronics, and antibacterial activity, N. Rauf *et al.* investigated a wide range of dopants, including rare earth metals, transition metals, noble metals, post-transition (poor) metals, and nonmetals. For instance, europium-doped ZnO has been explored for antibacterial activity, optical switching, and nonlinear optical (NLO) applications, while Zn–La–O systems co-doped with Cu have demonstrated enhanced antibacterial effects.^7^ N. Kangathara *et al.* used a straightforward coprecipitation approach to study pure ZnO and Mg-doped ZnO nanoparticles with average crystallite diameters of 29 and 33 nm, respectively. It was discovered that the Mg–ZnO nanostructures had an enhanced ethanol-sensing ability. Their increased active surface area and improved conductivity made them an attractive option for ethanol sensing applications.^8^ In order to improve and manage the multifunctional qualities of dysprosium-doped ZnO nanoparticles for optoelectronic and magnetoelectrical applications, N. Aggarwal *et al.* synthesised Dy-doped ZnO nanoparticles by a sol–gel method and used the four-probe method for analyzing resistivity dependence on the dopant concentration.^9^ A. Vanaja *et al.* reported that sol–gel-derived Cu-doped ZnO nanoparticles exhibited reduced particle size and improved stability. The peak intensity increased with copper doping, according to optical absorbance measurements, suggesting improved optical qualities, and because of their special qualities, ZnO nanoparticles were used in LEDs, varistors, UV absorbers, and the biomedical industry.^10^ After investigating yttrium-doped ZnO nanorods, D. Vaddi *et al.* reported that they retained the hexagonal wurtzite crystal structure and a 97% photodegradation efficiency at a 3% doping concentration.^11^ In their research on codoped ZnO nanoparticles, N. Lavanya *et al.* found that these particles had red-shifted optical characteristics and produced white light that was appropriate for LED applications.^12^ M. Bodiul Islam *et al.* concluded that with an increase in the Ag concentration, the band gap decreased, and the absorption edge of the NPs was red-shifted, indicating the insertion of a new energy level into the material's band structure. With 7% Ag doping, the lowest band gap (3.26 eV) was discovered. Moreover, Zn_0.93_Ag_0.07_O NPs demonstrated the strongest antibacterial activity, confirming their potential use as antibacterial agents.^13^

Zinc oxide nanoparticles doped with silver combine the antibacterial capabilities of silver with the characteristics of ZnO. ZnO NPs' antibacterial and antifungal properties can be strengthened by the addition of silver ions or particles.^14^ When Ag is doped into ZnO, possible changes in the band gap, which is connected to the material's optical absorption, and surface plasmon resonance, which influences the optical properties of the nanoparticles, may be seen.^15^

The problem of pollution resulting from dyes and the emergence of antibiotic-resistant microorganisms are becoming increasingly severe. Doped ZnO nanoparticles can significantly reduce the band gap. Doped nanoparticles are more effective than zinc oxide alone in combating germs and photocatalysis. Finding the ideal silver dopant concentration needed to produce devices with an appropriate degree of antibacterial activity remains a top research priority across a number of fields.

In this study, we aim to investigate zinc oxide nanoparticles and evaluate how doping influences their properties and performance. The duration of capping agent ethanol, addition and the incubation period were varied from previous studies to evaluate their impact on structural, optical, and crystallite size properties. A moderate incubation period favors smaller crystallite sizes. Further, we report a sol–gel method for the synthesis of Ag-doped ZnO nanoparticles, and the effect of different doping concentrations of silver on the antimicrobial and photocatalytic activity is studied.

## 2. Experimental

### 2.1 Synthesis of silver-doped zinc oxide nanoparticles

The sources of zinc, silver and oxygen were zinc acetate dehydrate [Zn(CH_3_COO)_2_·2H_2_O], silver nitrate [AgNO_3_], sodium hydroxide [NaOH], and ethanol [C_2_H_5_OH]. AR-grade chemicals were used immediately after purchase from Loba Chemicals. The precursor solutions were prepared in deionized water.

In order to obtain ZnO NPs, 100 mL of a 0.1 N Zn(CH_3_COO)_2_·2H_2_O solution was mixed with a 0.1 M NaOH solution by dropwise addition and stirred for two hours. For two hours, ethanol was gradually and dropwise added to the precursor mixture. The produced solution was incubated for 12 hours in order to form a gel. The gel was centrifuged and dried in an oven for the second time after being roasted at 60 °C for around four hours to produce a white solid. ZnO NPs were obtained by annealing the dry powder for two hours at 500 °C and labelled as S0.

**Fig. 1:**
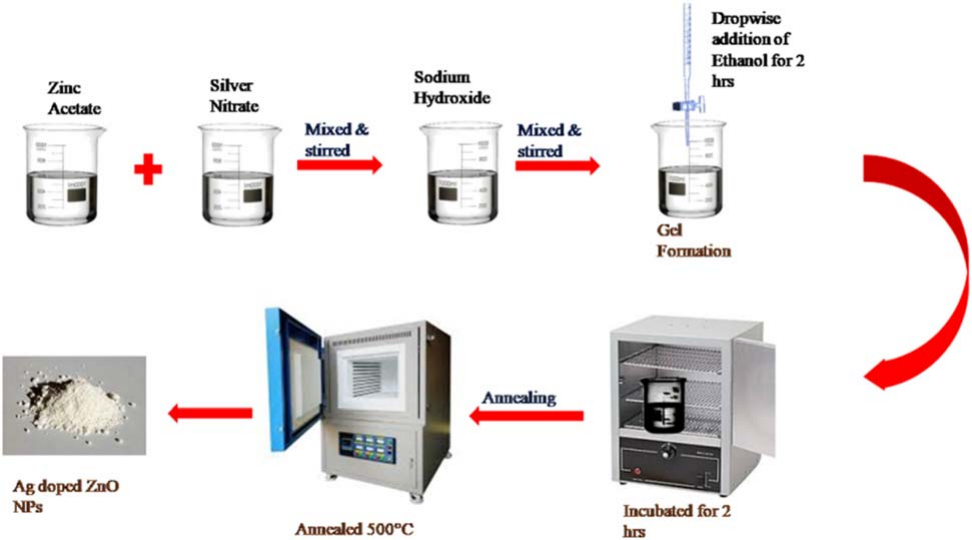
Schematic of the synthesis of Ag-doped ZnO NPs.

To synthesize 1, 3, 5, and 7 mol% Ag-doped ZnO NPs, 100 mL of a 0.1 N Zn(CH_3_COO)_2_·2H_2_O solution was added to the desired concentration of AgNO_3_ solution with constant stirring for about half an hour. The mixture was agitated to obtain a homogeneous solution. After that, a 0.1 M NaOH solution was gradually added to the mixture and stirred for two hours. For two hours, ethanol was gradually and dropwise added to the precursor mixture. The produced solution was incubated for 12 hours in order to form a gel. The gel was centrifuged and dried in an oven for the second time after being roasted at 60 °C for around four hours to produce a white solid. The samples labelled S1, S3, S5, and S7 represent 1, 3, 5, and 7 mol% Ag-doped ZnO NPs, respectively, which were obtained by annealing the dry powder for two hours at 500 °C. Fig. 1 depicts the schematic diagram of the synthesis of Ag-doped ZnO nanoparticles.

All experiments were done three times on different days to make sure the results were reliable.

### 2.2 Characterization techniques

All of the samples underwent X-ray diffraction (XRD) analytical measurements using the Ultima IV apparatus from Rigaku Corporation, Japan, which uses Cu-Kα radiation (*λ* = 1.5406 Å). A JEOL JSM-6360 was used to conduct the field-emission scanning electron microscopy (FESEM) and energy-dispersive X-ray spectroscopy (EDX) investigations. Using a JEOL JEM-2100 plus high-resolution transmission electron microscope (HRTEM), the morphology was analyzed. The Ag-doped ZnO NPs' UV-vis spectra were captured using a Shimadzu UV-2600i spectrometer (Japan). This spectrometer measured the absorbance edge in the wavelength range of 200–800 nm.

### 2.3 Photocatalytic activity test

This investigation evaluated the photocatalytic efficacy of ZnO (S0) and Ag-doped ZnO NPs (S1, S3, S5, and S7) *via* the utilization of methylene blue, a dye commonly utilized for such assessments. The examination was carried out to establish the capability of ZnO and Ag-doped ZnO NPs in effectively and consistently decomposing methylene blue (MB) at an ambient temperature. In a beaker of a moderate size, 100 mg of the catalyst (ZnO or Ag–ZnO) was mixed with 100 mL of a methylene blue solution with an initial concentration of 10 ppm under irradiation using a 36-W high-pressure mercury lamp positioned at a distance of 3 cm from the specimen.^16^ The catalyst mixture was subjected to magnetic stirring to achieve homogeneous dispersion of nanoparticles in the MB solution. The separation of the catalyst from the suspension was achieved through centrifugation, and UV-vis spectroscopic analysis was carried out using deionized water as the blank reference for quantifying the absorption of MB.

After a duration of 120 minutes comprising complete illumination, the transmittance of the solution containing MB was documented at 30-minute intervals and converted into absorbance. The extent of photocatalytic degradation was determined by measuring the absorbance of the solution at 664 nm. Eqn (1) was used to calculate the degradation efficiency of the dye.1
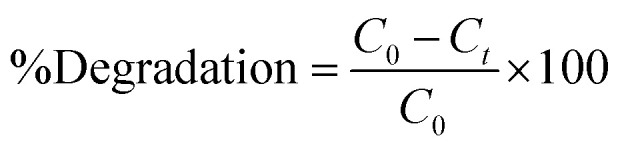


At the typical absorption wavelength of 664 nm, the initial absorbance was represented by *C*_0_, and the absorbance after *t* minute was represented by *C*_*t*_. The photodegradation efficiency was expressed as the highest photodegradation at each interval.^17^ The photodegradation rate constant, *k*, was determined by plotting log(*C*_*t*/_*C*_0_) against time on a line graph.

### 2.4 Antibacterial studies

The study of the antimicrobial activity of S0, S1, S3, S5 and S7 was performed against Gram-positive bacteria *Staphylococcus aureus*, Gram-negative bacteria *Pseudomonas aeruginosa* and fungi *Candida albicans*.^18^ The microorganism's inoculum was derived from the bacterial cultures. 15 mL of a nutrient agar (HiMedia) medium was added to sterile Petri plates and allowed to cool and solidify. 100 µL of a bacterial strain broth was aspirated, evenly spread over the medium using a spreading rod and dried thoroughly. Following agar solidification, the samples were positioned on the plates accordingly and incubated at 37 °C for 24 hours. The assessment of antibacterial activity was conducted by measuring the diameters of the zones of inhibition (ZIs).

Each compound was dissolved in DMSO at a desired concentration and kept in the fridge until needed for the antifungal study. The compounds were tested for their antifungal properties using the agar well diffusion assay.^19,20^ Sabouraud dextrose agar from HiMedia was utilized to cultivate fungi. A solution with an acidic pH (pH 5.5–5.6) and a high concentration of glucose (40%) was made by combining Sabouraud dextrose agar (SDA) with distilled water, followed by sterilizing it at 120 °C for 15 minutes. 25 mL of an SDA medium at 45 °C was safely added to each sterile Petri dish measuring 100 mm × 15 mm. To determine the number of fungi spores, they were mixed with normal saline to reach a volume of 1 mL, and then, they were counted using a haemocytometer (Neubauer chamber). After the agar had solidified, sterile cork borers were used to create 8-mm wells. Next, 0.1 mL (100 µL) of each stock solution containing compounds with the final concentrations was added to each well, and the plates were then incubated at 29 °C for 24 hours.

Each Petri dish had two wells treated with DMSO and positive and negative controls, respectively, of the reference antifungal medication, clotrimazole (1 mg mL^−1^), dissolved in DMSO. The diameter (mm) of the clear zone of growth inhibition was used as a measure of antifungal activity.^21,22^

## 3. Results and discussion

### 3.1 X-ray diffraction measurements

**Fig. 2:**
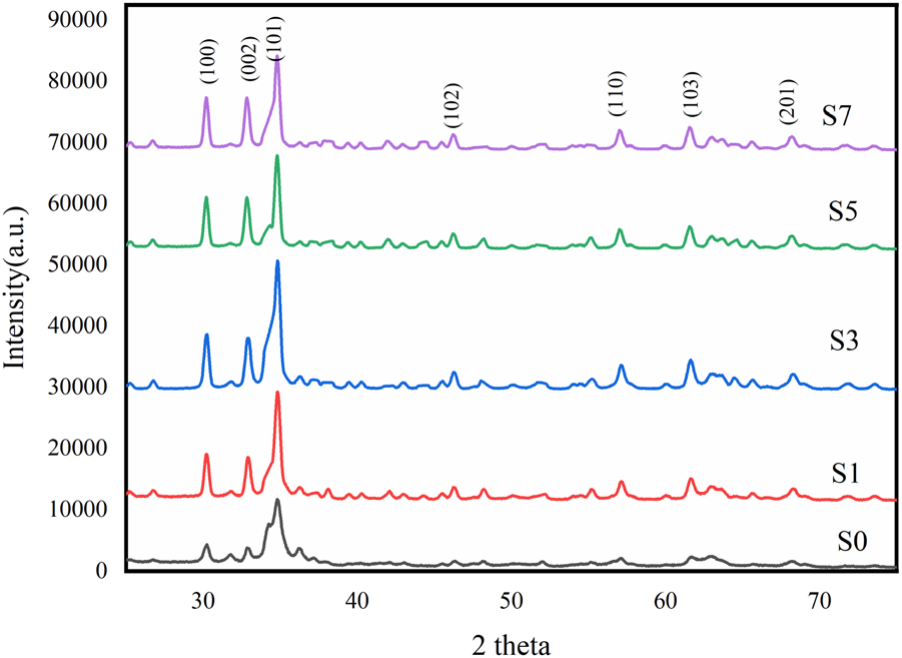
The XRD patterns of Ag-doped ZnO with different doping concentrations of silver (1, 3, 5 and 7 mol%).

The crystalline structure and phase structure variations among S0, S1, S3, S5 and S7 were assessed using XRD analysis, as displayed in Fig. 2. An XRD scan covering the range from 20° to 80° was selected to analyze crystalline phases, lattice parameters, and phase identification. The broadened peaks observed in the XRD pattern indicate the successful formation of nanocrystalline structures.^23^ The XRD patterns of all samples were obtained at angles between 30° and 80°. The peaks representing the (100), (002), (101), (102), (110), and (103) planes are observed at the 2*θ* values of 31.80°, 34.45°, 36.25°, 47.62°, 56.69°, and 62.95°, respectively. No additional peaks were observed, and all diffraction peaks were indexed to the hexagonal wurtzite structure of ZnO, confirming the absence of any secondary phases, in agreement with JCPDS card no. 01-079-2205.^24^

If Zn^2+^ ions are replaced by silver, an equivalent peak shift in the XRD pattern is anticipated. The absence of this shift in the recorded XRD suggests that Ag particles have aggregated at the ZnO grain boundaries or that the amount of Ag integrated at the substitutional Zn site is negligible. The difference in the ionic radii of Ag^+^ (1.22 Å) and Zn^2+^ (0.72 Å) makes the latter possibility implausible, as silver particles preferentially choose to aggregate near the ZnO grain boundaries. There are no metallic Ag peaks visible in the Ag–ZnO's XRD. The low amount of Ag could be the cause of this. It is improbable that the prepared Ag–ZnO contains Ag as Ag_2_O. No additional secondary phases such as Ag_2_O, Zn or Ag metallic phases are observed in all the samples.

Doping Ag into ZnO NPs increases the crystallinity, as we observed. The XRD peaks of Ag-doped ZnO exhibit a slight shift toward lower diffraction angles. Because of lattice distortion, the strength of the diffraction peaks diminishes with peak broadening as the silver doping content rises.^25^ The Debye–Scherrer formula was used for calculating the particle size (eqn (2)). The XRD peaks of Ag-doped ZnO exhibit a slight shift toward lower diffraction angles. As the Ag doping concentration increases, the crystallinity of the produced nanoparticles increases. Additionally, it is noted that when the Ag concentration increases when doping ZnO, the average crystallite size increases from 22 to 31 nm. The little amount of silver absorbed on the ZnO surface, the separation of Ag ions at the ZnO boundaries, and the lower solubility of Ag in ZnO could collectively be the cause of this.^26,27^2
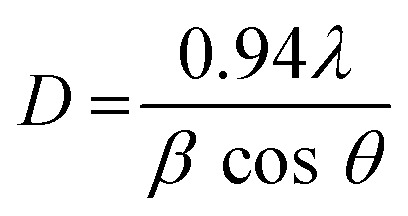
where *λ* is the wavelength of the incident Cu Kα beam (*λ* = 1.5405 Å), *θ* is the Bragg angle in radians, and *β* is the full width at half maximum (FWHM) of the peak. It is also observed that the average crystallite size increases from 22 to 31 nm with increasing Ag doping in ZnO (Table 1).

**Table 1:** Crystallite size of ZnO and Ag-doped ZnO nanoparticles

Sample	Crystallite size (nm)
S0	22 ± 3.45
S1	25 ± 1.83
S3	26 ± 3.77
S5	28 ± 6.74
S7	31 ± 8.80

### 3.2 FESEM and EDX analysis

**Fig. 3:**
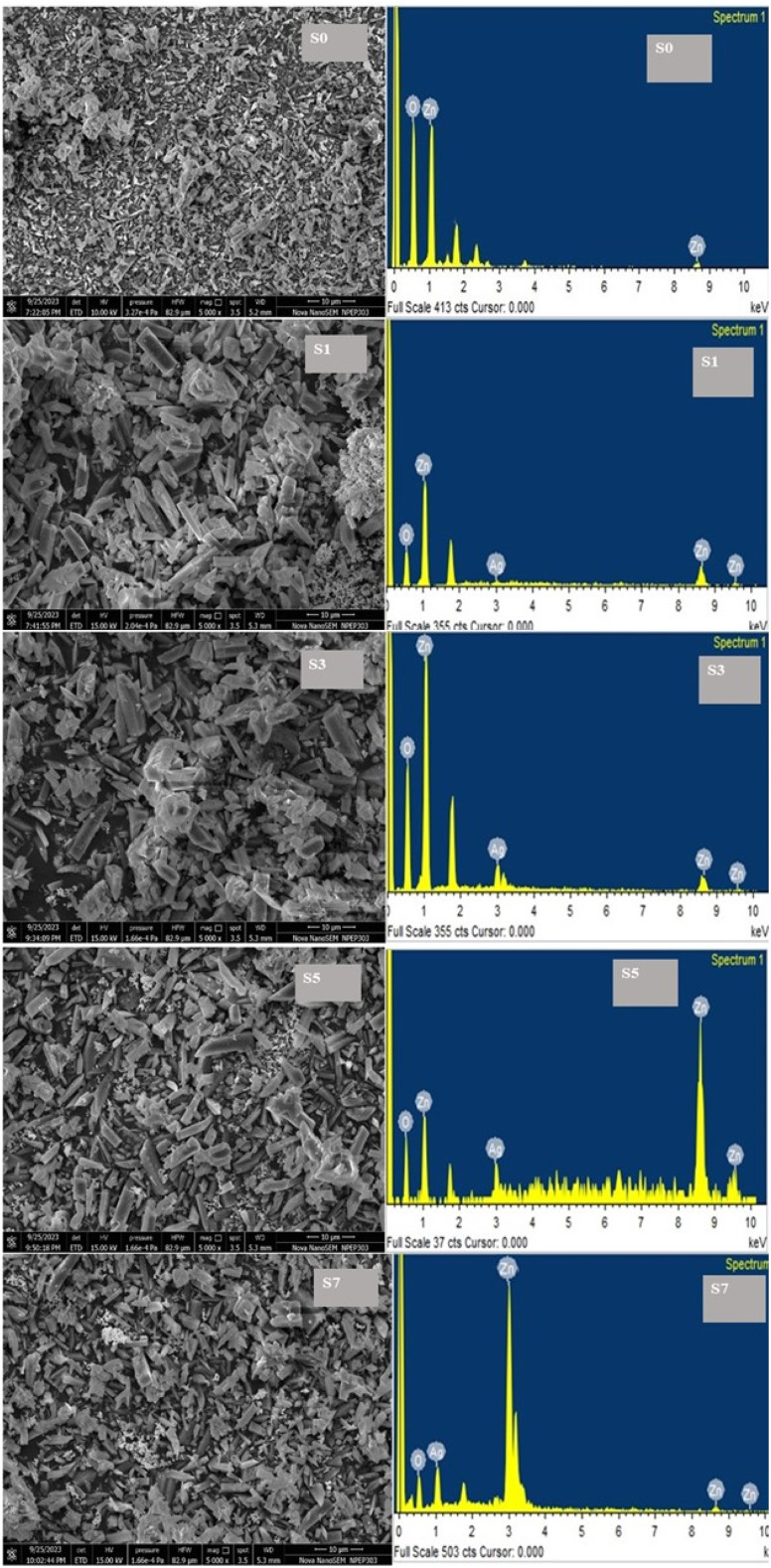
FESEM and EDS images of undoped ZnO (S0) and Ag-doped ZnO (S1, S3, S5, and S7).

It has been demonstrated that ZnO NPs' morphological characteristics are significantly affected by silver doping. Field-emission scanning electron microscopy (FESEM) was utilized to investigate the impact of Ag doping on ZnO NPs, specifically the way in which the addition of Ag modifies ZnO's morphology. Fig. 3 shows the morphologies of ZnO (for a comparative study) and Ag-doped ZnO at different molar concentrations. Both pure ZnO and Ag-doped ZnO NP samples consist of densely packed columns that form nanoparticles. The analysis of the FESEM micrographs reveals a distinct alteration in the morphology of ZnO upon silver doping. The FESEM images of Ag-doped ZnO demonstrate a more organized morphology, with reduced particle agglomerations compared to ZnO NPs. The presence of silver may have disrupted the growth mechanism, leading to the development of uniformly dispersed nano-sized particles and resulting in a smoother surface morphology, as observed in previous studies.^28^ An increase in the concentration of silver doping could potentially bring about a noticeable shift in the size distribution of the nanoparticles.^29^ Higher levels of silver ions might accelerate nucleation rates and promote particle growth, thereby leading to the formation of larger nanoparticles.^30^Fig. 3 shows the FESEM images of samples prepared with varying concentrations of silver, ranging from 0 to 7 mol%. The influence of Ag on the size of ZnO NPs is clearly depicted in the images. In Fig. 3 (S0), which presents 0% Ag, a mixture of small and large particles is observed. Upon the addition of 1% Ag, all grains appear small with an increased surface/volume ratio. Further increments in the amount of Ag result in a reduction in the number of small particles, which nearly disappear in the samples containing 7% Ag. Consequently, an increase in the quantity of Ag leads to variations in the particle size, causing a slight decrease in the surface/volume ratio. At higher concentrations of silver doping, the surface morphology may exhibit greater roughness or irregularity due to the presence of additional silver ions and their impact on the dynamics of crystal growth. The EDX spectrum is a commonly employed analytical technique for assessing the elemental composition of materials, as shown in Table 2. Fig. 3 provides vital information on the distribution and presence of silver in the ZnO matrix. The intensity of these peaks enables the determination of the relative concentrations of each element. The presence and concentration of silver in the ZnO matrix were confirmed by analyzing the intensity of the Ag peaks. Noteworthy, peaks associated with Zn and O signify the presence of the ZnO matrix. The variations in the percentage corresponding to the doping level are confirmed by the intensity of Ag peaks.

**Table 2:** EDS elemental composition analysis

Elements	S0		S1		S3		S5		S7	
	Weight %	Atomic %	Weight %	Atomic %	Weight %	Atomic %	Weight %	Atomic %	Weight %	Atomic %
O K	50.92 ± 0.2	79.53 ± 0.2	31.1 ± 0.2	65.15 ± 0.2	37.99 ± 0.2	72.88 ± 0.2	39.07 ± 0.2	75.4 ± 0.2	41.42 ± 0.2	78.31 ± 0.2
Zn L	49.09 ± 0.2	20.47 ± 0.2	66.54 ± 0.2	34.11 ± 0.2	51.22 ± 0.2	24.05 ± 0.2	38.45 ± 0.2	18.16 ± 0.2	29.61 ± 0.2	13.57 ± 0.2
Ag L	—	—	2.36 ± 0.2	0.73 ± 0.2	10.79 ± 0.2	3.07 ± 0.2	22.48 ± 0.2	6.43 ± 0.2	28.97 ± 0.2	8.11 ± 0.2

### 3.3 HRTEM analysis

**Fig. 4:**
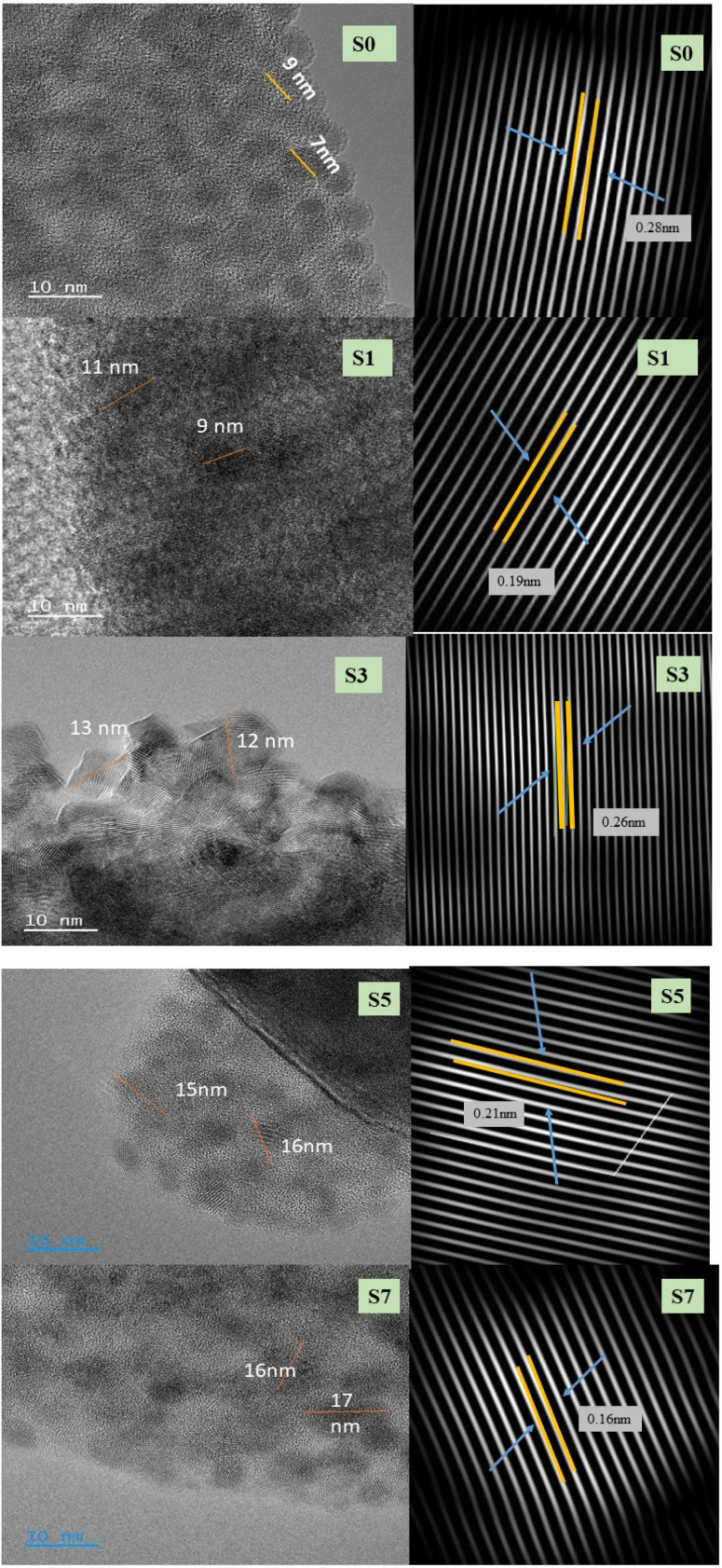
HRTEM images of undoped ZnO (S0) and Ag-doped ZnO (S1, S3, S5, and S7) along with the corresponding FFTs and *d*-spacings.

The effect of the doping concentration was examined using HRTEM analysis. The high-resolution transmission emission microscopy images of ZnO and Ag-doped ZnO NPs (S0, S1, S3, S5 and S7) are shown in Fig. 4. The agglomerated Ag particles within the ZnO nanoparticles exhibit a rod-like morphology. This size fluctuation suggests that Ag within the ZnO crystals has introduced lattice stress.^31^ Additionally, the images clearly show the edges of distinct planes, demonstrating the nanoparticles' single-crystalline structure. Furthermore, the growth direction and lattice spacing are shown in Fig. 4, along with the fast Fourier transform (FFT) and inverse fast Fourier transform (IFFT) patterns from the same region.

The pristine ZnO nanoparticles demonstrate a measured separation of 2.8 Å ° between neighboring fringes, which corresponds effectively with the interplanar distance of the 100 plane.^32^ Moreover, the fast Fourier transform (FFT) pattern indicates that the growth direction is 100, illustrating a vertical growth parallel to the *c*-axis. The inverse fast Fourier transform (IFFT) patterns of the samples containing 1, 3, 5, and 7 mol% Ag reveal fringe spacings of 1.9 Å, 2.6 Å, 2.1 Å, and 1.6 Å, respectively. These measurements closely match the *d* spacings of the (102), (002), (102), and (110) planes, respectively, in accordance with the growth direction observed in the FFT patterns.^33^

### 3.4 Optical properties

**Fig. 5:**
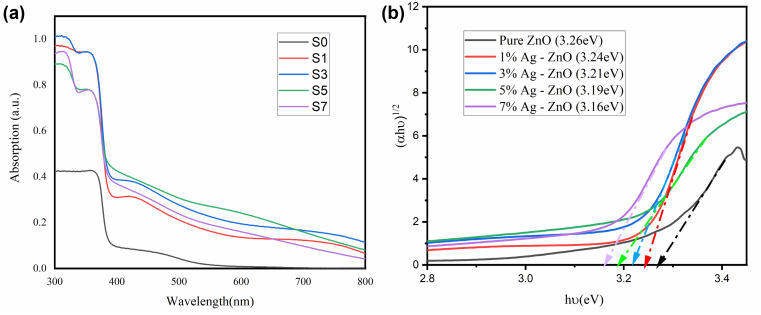
(a) UV-vis spectra of Ag-doped ZnO nanoparticles. (b) Band gap of Ag-doped ZnO.

UV-visible spectroscopic investigations were performed in order to figure out how the optical properties of ZnO NPs were affected by Ag doping. Fig. 5a demonstrates the UV-vis spectra of the ZnO and Ag-doped ZnO NPs (S0, S1, S3, S5 and S7). Furthermore, for comparison, the spectrum of the ZnO nanoparticles (containing 0 mol% Ag) is presented. A prominent shoulder can be seen in the spectra for each sample in the 350–400 nm wavelength region.^34^ According to this phenomenon, Ag-doped ZnO NPs' intrinsic band gap represents electron transitions from the valence band to the conduction band. Ag-doped ZnO catalysts exhibit increased absorption in the visible spectrum mainly as a result of metallic silver nanoparticle-induced surface plasmon resonance (SPR) and a decrease in ZnO's band gap energy.

Using eqn (3), the optical band gaps (*E*_g_) of the pure ZnO and Ag-doped ZnO NPs were calculated by extrapolating the linear region of the *α*^2^*vs. hv* plots.

3 (*αhν*)^2^ = *A*(*hν* − *E*_g_)^*n*^

where *α*, *hν*, *A*, and *E*_g_ are the absorption coefficient, photon energy, relation constant, and optical band gap, respectively. The best fit for these NPs is obtained at *n* = 1, when a notable linearity is seen, indicating a direct permitted transition.^35^ The band gap values of the S0, S1, S3, S5, and S7 samples are measured to be 3.26 eV, 3.24 eV, 3.21 eV, 3.19 eV, and 3.18 eV, respectively, which are listed in Table S1. An increase in the Ag doping concentration in ZnO NPs reduces the band gap and leads to a red-shift in the absorption edge position, which can be elucidated by the substitution of Ag^+^ ions for Zn^2+^ ions within the ZnO lattice, thereby counteracting the influence of the particle size.^36^ The shift in the absorption edge also signifies variations in the direct band gap of the nanoparticles (Table S1).

Moreover, the Ag clusters' Fermi level is traversed by the conduction band (CB) electrons that are energized from the valence band (VB), and the electron transport will proceed until the whole Ag Fermi level and ZnO CB value equalize.^37^ It is reasonable to anticipate increased electron transport and decreased *E*_g_ values as the Ag content rises. As a result, increasing the Ag concentration promotes the formation of more oxygen vacancies, leading to a reduction in the band gap (*E*_g_) value.

By contrast, the Burstein–Moss effect causes band gap widening when the number of electron donors in the system increases.^38^ This results in a blue-shift in the band gap of ZnO, which can be attributed to electron donors like Al and Ga, and Fig. 5b illustrates how adding electron acceptors such as Ag reduces the band gap of ZnO NPs.

### 3.5 Photocatalytic analysis

**Fig. 6:**
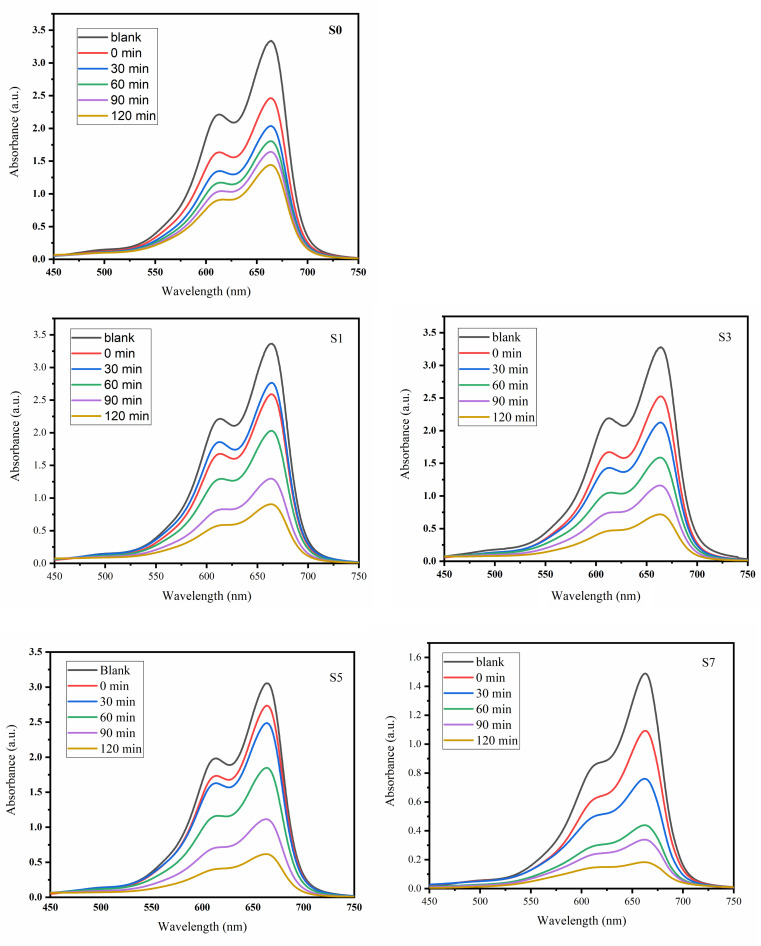
UV-visible absorption patterns of the MB dye examined using ZnO and Ag-doped ZnO samples (S0, S1, S3, S5 and S7).

The photodegradation of MB dye was studied using the degradation of a 10 ppm aqueous solution under 36-W UV light irradiation every 30 minutes using the S0, S1, S3, S5, and S7 catalysts. This study's main objective was to ascertain how the concentration of Ag doping in ZnO NPs affects the breakdown of a pollutant, such as MB dye. The UV-vis absorption spectra were obtained at wavelengths ranging from 300 to 700 nm. Fig. 6 shows the time-dependent absorbance spectra for 120 minutes under UV light irradiation. The plot indicates that the characteristic absorption peak (*λ*_max_) of MB is observed at 664 nm. Ag-doped ZnO NPs exhibits notable photodegradation after 120 minutes. Based on MB's absorbance at 664 nm, eqn (1) was utilized to calculate the degradation efficiency of MB after 120-min UV exposure. The enhancement in the photocatalytic activity of semiconductor materials can be attributed to multiple causes. The electron transfer from the dye to the CB of ZnO and Ag's Fermi level, which also simultaneously participates in electron transfer from ZnO's CB, is the basis for the mechanism of photocatalytic reactions in the presence of a dye.^39^ Therefore, in the presence of both Ag–ZnO nanoparticles and dye molecules, the interaction of conduction band (CB) electrons with dissolved oxygen in the solution results in the generation of reactive oxygen species. The dye degradation is caused by these active oxygen species, and their quantity increases with the Ag concentration.^40^ Moreover, the enhanced photocatalytic efficiency can be attributed to oxygen vacancies, which promote the transfer of photoexcited electrons to the dye.^41^ Based on these discussions, it can be said that in our instance, the ZnO nanoparticles' photocatalytic activity increases with the amount of Ag present, which causes an increase in oxygen defects. This outcome is consistent with the recognized processes that increase the Ag-doped ZnO NPs' capacity for photocatalysis.

The saturation of photocatalytic activity has been achieved, but this phenomenon can be interpreted in multiple ways. The presence of metallic nanoparticles of Ag in the sample may initially lead to a reduction in the available free surface area for interaction with light and adsorption of dye molecules by ZnO. However, the proximity of electric charge carriers to the surface of the particle, particularly in the scenario of particles with an extremely minute size, may result in an increased likelihood of recombination occurring. This recombination process can then surpass interfacial charge transfer, leading to a decline in the photocatalytic activity of the particles. The results obtained indicate that achieving enhanced photocatalytic activity depends on the specific particle size and the optimal amount of Ag doping in ZnO.^42^

**Fig. 7:**
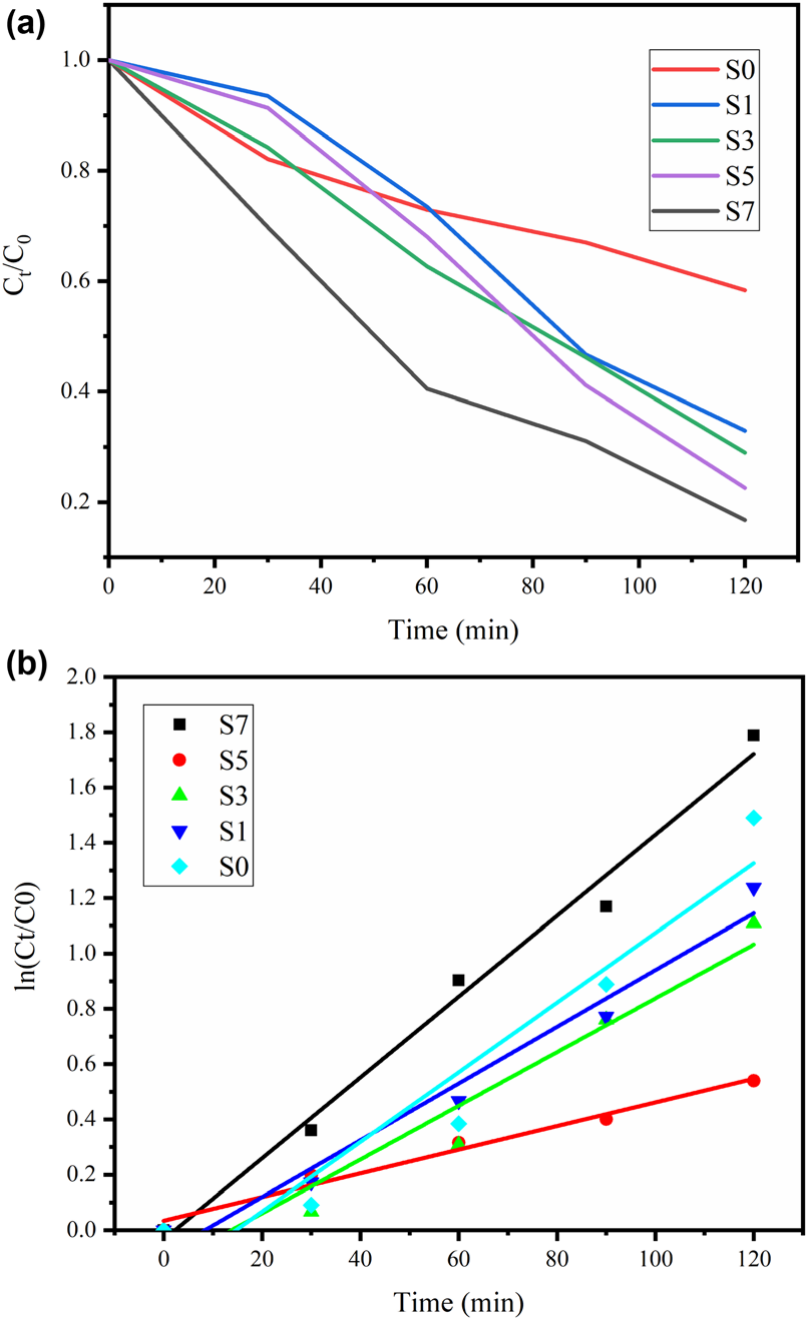
(a) Photodegradation plots of MB dye over ZnO and Ag-doped ZnO catalysts, represented as *C*_*t*_/*C*_0_*versus* time. (b) Kinetic plots of MB dye degradation over ZnO and Ag-doped ZnO catalysts, expressed as ln(*C*_*t*_/*C*_0_) *versus* time.

In summary, the Ag doping of ZnO NPs results in increased photocatalytic activity in the visible and UV regions of the electromagnetic spectrum, at least for the effective removal of MB dye. The *C*_*t*_/*C*_0_*vs.* irradiation time curves of Ag-doped ZnO NPs are shown in Fig. 7a. The variation of ln(*C*_*t*_/*C*_0_) *vs.* irradiation time for the prepared catalysts is shown in Fig. 7b. The rate constants are of the first order and found to be 0.00427, 0.00971, 0.01026, 0.01258 and 0.01462 for S0–S7, respectively. Based on a comprehensive analysis, ZnO and Ag-doped ZnO catalysts degrade MB with optimal values of 69% 73%, 77%, 80% and 88% for S0, S1, S3, S5 and S7, respectively, as presented in Fig. 7a and b. A comparative study with previously reported research articles is shown in Table S2.^43–46^Ag + *hυ* → h^+^ + e^−^Ag(e^−^) + ZnO → ZnO(e_CB_^−^) + Ag(h^+^)ZnO(e_CB_^−^) + O_2_ → ZnO + O_2_^·−^ → 2HO → H_2_O_2_ → HO + OH^−^ + O_2_Ag(h^+^) + OH^−^ → Ag + HO·OH + dye → dye_ox_ → intermediate → CO_2_ + H_2_O

### 3.6 Antimicrobial activity

The well diffusion method, a common laboratory procedure to assess an antimicrobial agent's efficacy against bacteria, was used to test the antibacterial activity of these nanoparticles (Fig. S1).

Using this method, the materials to be tested, *i.e.*, ZnO and Ag-doped ZnO NPs, were put in wells created in an agar plate that was infected with bacteria. Following incubation, the zone of inhibition—the region surrounding the wells in which bacteria cannot proliferate—was measured.

For testing, two widely used bacterial strains were employed*. S. aureus* is a Gram-positive bacterium that is distinguished by its thick peptidoglycan cell wall. Gram-negative bacteria include *P. aeruginosa*, which has an extra outer membrane but a thinner peptidoglycan layer. These two varieties reflect distinct bacterial cell wall architectures, which frequently affect an organism's vulnerability to antimicrobial agents.

**Fig. 8:**
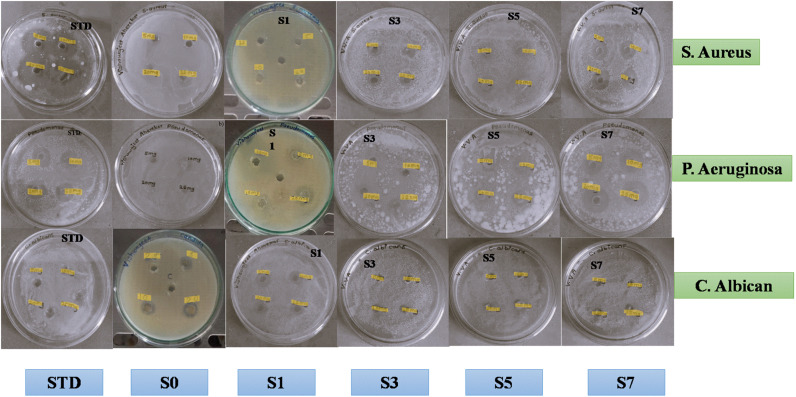
Antibacterial and antifungal activity of Ag-doped ZnO NPs against *S. aureus*, *P. aeruginosa* and *C. albicans*.

ZnO and Ag-loaded ZnO compounds were added to the culture medium that was preinoculated with the microbial suspension, and after a 24-hour incubation period, the clear zone of growth inhibition was measured. The cells of fungi were distorted by the nanoparticles, which interfere with cellular activity and prevent the bacteria from developing.^47^ Reactive oxygen species production increases the porousness of cell membranes, facilitating cell death.^48^ The antibacterial and antifungal activities of S0, S1, S3, S5 and S7 are shown in Fig. 8. Because of the variation in the membrane's ROS sensitivity and thickness, ZnO is more prone to bacterial growth. However, delayed oxidation causes silver, a corrosion-resistant noble metal, to release ions, which interact with bacterial cell membranes to produce antibacterial characteristics.^49^

The effectiveness of nanoparticles in destroying germs is significantly influenced by their silver content. The addition of silver causes zinc oxide to produce more electron–hole pairs, which enhances oxidative stress in microorganisms and damages their cells.^50^ On the other hand, excessive silver may lead to agglomeration or adversely affect the host system, thereby reducing its efficiency, whereas lower silver concentrations may be insufficient to eliminate resistant microorganisms.

## 4. Conclusion

In the present study, ZnO and Ag-doped ZnO NPs are synthesized by a sol–gel method. The optical band gap is found to decrease from 3.26 eV for pure ZnO NPs to 3.18 eV for 7 mol% Ag-doped ZnO NPs. XRD analysis indicates a hexagonal wurtzite structure, and the average crystallite size increases from 22 nm for pure ZnO NPs to 31 nm for 7 mol% Ag-doped ZnO NPs. The FESEM study indicates a polycrystalline nature, and HRETM analysis confirms the growth of particles along the respective planes at various Ag doping levels. The antibacterial activity analysis of Ag-doped ZnO NPs reveals that they are more specific towards bacteria *S. aureus* and *P. aeruginosa* than fungi *C. albicans.* As the Ag content in NPs increases, the photocatalytic activity towards MB dye increases, which is almost 88% for 7% Ag–ZnO NPs.

Research on doping levels and microorganisms is limited. It mainly focuses on a single case of dye degradation. We need more studies on different types of microbes, a broader range of pollutants, stability, and possible cytotoxicity to fully understand their biomedical and environmental uses. Future research is focused on developing innovative approaches, including the advancement of nanomedicine for targeted cancer therapy and the disruption of bacterial communication, along with the use of phytochemical-based synthesis methods.

## Author contributions

Conceptualization, methodology and investigation: Vishwajeet. V. Aherkar; software and validation: Anmar a. Al-shimary; formal analysis and data curation: Ali Abdulmawjood Mohammed; resources: Hiba Qasim Mahmoud and Bilal Husam Jasim; supervision: Rekha M. Ovhal and Panchsheela Ubale; writing – original draft preparation: Vishwajeet V. Aherkar; and writing – review and editing: Panchsheela Ubale.

## Conflicts of interest

Regarding the content of this article, the authors declare that they have no conflicts of interest.

## Data Availability

Data will be available on request.

## References

[cit1] Ikoma H. , Semiconductors, Impurity and Defect States, in. Encyclopedia of Condensed Matter Physics, 2024pp. 516–519, 10.1016/B978-0-323-90800-9.00288-2

[cit2] Maddahi P., Shahtahmasebi N., Kompany A., Mashreghi M., Safaee S., Roozban F. (2014). Effect of doping on structural and optical properties of ZnO nanoparticles: study of antibacterial properties. Mater. Sci..

[cit3] Khan S., Stamate E. (2022). Comparative Study of Aluminum-Doped Zinc Oxide, Gallium-Doped Zinc Oxide and Indium-Doped Tin Oxide Thin Films Deposited by Radio Frequency Magnetron Sputtering. Nanomaterials.

[cit4] Bharat T. C., Shubham, Mondal S., Gupta H. S., Singh P. K., Das A. K. (2019). Synthesis of Doped Zinc Oxide Nanoparticles: A Review. Mater. Today Proc..

[cit5] Kumar S. G. , Kavitha R. and Sushma C., Chapter 9 - Doped zinc oxide nanomaterials: structure–electronic properties and photocatalytic applicationsa, in, Surf. Sci. Photocatal., ed, J. Yu, M. Jaroniec, C. Jiang, Elsevier, 2020, pp. 285–312. 10.1016/B978-0-08-102890-2.00009-9

[cit6] Mondloch J. E., Bayram E., Finke R. G. (2012). A review of the kinetics and mechanisms of formation of supported-nanoparticle heterogeneous catalysts. J. Mol. Catal. A:Chem..

[cit7] Rauf N. (2023). Recent Progress of ZnO-Based Nanoparticle: Synthesizing Methods of Various Dopant and Applications. J. Fis. Flux J. Ilm. Fis. FMIPA.

[cit8] Kangathara N., Sabari V., Saravanan L., Elangovan S. (2022). Synthesis, Characterization, and Comparison of Pure Zinc Oxide and Magnesium-Doped Zinc Oxide Nanoparticles and their Application on Ethanol Sensing Activities. J. Nanomater..

[cit9] Aggarwal N., Vasishth A., Kaur K., Verma N. K. (2020). Nanoscale Properties of Dysprosium-Doped Zinc Oxide Nanoparticles. J. Supercond. Novel Magn..

[cit10] Vanaja A., Suresh M., Jeevanandam J., Venkatesh, Gousia S., Pavan D., Balaji D., Murthy N. B. (2019). Copper-Doped Zinc Oxide Nanoparticles for the Fabrication of white LEDs. Prot. Met. Phys. Chem. Surf..

[cit11] Vaddi D. R., Vinukonda K., Patnala R. K., Kanithi Y., Gurugubelli T. R., Bae J., Koutavarapu R., Lee D.-Y., Shim J. (2023). Effect of yttrium doping on the crystal structure, optical, and photocatalytic properties of hydrothermally synthesized ZnO nanorods. Mater. Sci. Eng., B.

[cit12] Lavanya N., Deepak N. K. (2023). Excitation wavelength altered PL study of Co doped ZnO nanoparticles suitable for white LED application. Z. Naturforsch., A:Phys. Sci..

[cit13] Islam M. B., Haque M. J., Shehab N. M., Rahman M. S. (2023). Synthesis and characterization (optical and antibacterial) of silver doped zinc oxide nanoparticles. Open Ceram..

[cit14] Karunakaran V. R. C., Gomathisankar P. (2011). Photodeposited Surface Ag on ZnO Nanocrystals and the Optical, Electrical, Photocatalytic, and Bactericidal Properties. Synth. React. Inorg., Met.-Org., Nano-Met. Chem..

[cit15] Han Z., Ren L., Cui Z., Chen C., Pan H., Chen J. (2012). Ag/ZnO flower heterostructures as a visible-light driven photocatalyst via surface plasmon resonance. Appl. Catal. B Environ..

[cit16] Elshahawy M. F., Ahmed N. A., Mohamed R. D., Ali A. E.-H., Raafat A. I. (2023). Radiation synthesis and photocatalytic performance of floated graphene oxide decorated ZnO/alginate-based beads for methylene blue degradation under visible light irradiation. Int. J. Biol. Macromol..

[cit17] Li F. B., Li X. Z. (2002). The enhancement of photodegradation efficiency using Pt–TiO2 catalyst. Chemosphere.

[cit18] Matuschek E., Brown D. F. J., Kahlmeter G. (2014). Development of the EUCAST disk diffusion antimicrobial susceptibility testing method and its implementation in routine microbiology laboratories. Clin. Microbiol. Infect..

[cit19] Ashok U. P., Kollur S. P., Anil N., Arun B. P., Jadhav S. N., Sarsamkar S., Helavi V. B., Srinivasan A., Kaulage S., Veerapur R., Al-Rashed S., Syed A., Ortega-Castro J., Frau J., Flores-Holguín N., Glossman-Mitnik D. (2020). Preparation, Spectroscopic Characterization, Theoretical Investigations, and In Vitro Anticancer Activity of Cd(II), Ni(II), Zn(II), and Cu(II) Complexes of 4(3H)-Quinazolinone-Derived Schiff Base. Molecules.

[cit20] Boyanova L., Gergova G., Nikolov R., Derejian S., Lazarova E., Katsarov N., Mitov I., Krastev Z. (2005). Activity of Bulgarian propolis against 94 Helicobacter pylori strains in vitro by agar-well diffusion, agar dilution and disc diffusion methods. J. Med. Microbiol..

[cit21] Chandrasekaran M., Venkatesalu V. (2004). Antibacterial and antifungal activity of Syzygium jambolanum seeds. J. Ethnopharmacol..

[cit22] Ubale P., Mokale S., More S., Waghamare S., More V., Munirathinam N., Dilipkumar S., Das R. K., Reja S., Helavi V. B., Kollur S. P. (2022). Evaluation of in vitro anticancer, antimicrobial and antioxidant activities of new Cu(II) complexes derived from 4(3H)-quinazolinone: Synthesis, crystal structure and molecular docking studies. J. Mol. Struct..

[cit23] Bindu P., Thomas S. (2014). Estimation of lattice strain in ZnO nanoparticles: X-ray peak profile analysis. J. Theor. Appl. Phys..

[cit24] Das J., Khushalani D. (2010). Nonhydrolytic Route for Synthesis of ZnO and Its Use as a Recyclable Photocatalyst. J. Phys. Chem. C.

[cit25] Pal M., Pal U., Jiménez J. M. G. Y., Pérez-Rodríguez F. (2012). Effects of crystallization and dopant concentration on the emission behavior of TiO2:Eu nanophosphors. Nanoscale Res. Lett..

[cit26] Ashebir M. E., Tesfamariam G. M., Nigussie G. Y., Gebreab T. W. (2018). Structural, Optical, and Photocatalytic Activities of Ag-Doped and Mn-Doped ZnO Nanoparticles. J. Nanomater..

[cit27] Rekha K., Nirmala M., Nair M. G., Anukaliani A. (2010). Structural, optical, photocatalytic and antibacterial activity of zinc oxide and manganese doped zinc oxide nanoparticles. Phys. B Condens. Matter.

[cit28] Pugazhenthiran N., Sathishkumar P., Albormani O., Murugesan S., Kandasamy M., Selvaraj M., Suresh S., Kumar S. K., Contreras D., Váldes H., V Mangalaraja R. (2023). Silver nanoparticles modified ZnO nanocatalysts for effective degradation of ceftiofur sodium under UV-vis light illumination. Chemosphere.

[cit29] Kayani Z. N., Manzoor F., Zafar A., Mahmood M., Rasheed M., Anwar M. (2020). Impact of Ag doping on structural, optical, morphological, optical and photoluminescent properties of ZnO nanoparticles. Opt. Quant. Electron..

[cit30] Liu Y., Tai K., Dillon S. J. (2013). Growth Kinetics and Morphological Evolution of ZnO Precipitated from Solution. Chem. Mater..

[cit31] Macková A., Jagerová A., Malinský P., Cutroneo M., Flaks J., Nekvindová P., Michalcová A., Holý V., Košutová T. (2020). Nanostructures in various Au ion-implanted ZnO facets modified using energetic O ions. Phys. Chem. Chem. Phys..

[cit32] Zhu Y., Zhou Y. (2008). Preparation of pure ZnO nanoparticles by a simple solid-state reaction method. Appl. Phys. A.

[cit33] Thool G. S., Singh A. K., Singh R. S., Gupta A., Susan M. A. B. H. (2014). Facile synthesis of flat crystal ZnO thin films by solution growth method: A micro-structural investigation. J. Saudi Chem. Soc..

[cit34] Phuruangrat A., Mad-ahin S., Yayapao O., Thongtem S., Thongtem T. (2015). Photocatalytic degradation of organic dyes by UV light, catalyzed by nanostructured Cd-doped ZnO synthesized by a sonochemical method. Res. Chem. Intermed..

[cit35] Moharram A. H., Mansour S. A., Hussein M. A., Rashad M. (2014). Direct Precipitation and Characterization of ZnO Nanoparticles. J. Nanomater..

[cit36] Mosquera E., Rojas-Michea C., Morel M., Gracia F., Fuenzalida V., Zárate R. A. (2015). Zinc oxide nanoparticles with incorporated silver: Structural, morphological, optical and vibrational properties. Appl. Surf. Sci..

[cit37] Kumar S. G., Rao K. S. R. K. (2017). Comparison of modification strategies towards enhanced charge carrier separation and photocatalytic degradation activity of metal oxide semiconductors (TiO 2 , WO 3 and ZnO). Appl. Surf. Sci..

[cit38] Gibbs Z. M., LaLonde A., Snyder G. J. (2013). Optical band gap and the Burstein–Moss effect in iodine doped PbTe using diffuse reflectance infrared Fourier transform spectroscopy. New J. Phys..

[cit39] Ravishankar T. N., Manjunatha K., Ramakrishnappa T., Nagaraju G., Kumar D., Sarakar S., Anandakumar B. S., Chandrappa G. T., Reddy V., Dupont J. (2014). Comparison of the photocatalytic degradation of trypan blue by undoped and silver-doped zinc oxide nanoparticles. Mater. Sci. Semicond. Process..

[cit40] Wang J., Guo Y., Liu B., Jin X., Liu L., Xu R., Kong Y., Wang B. (2011). Detection and analysis of reactive oxygen species (ROS) generated by nano-sized TiO2 powder under ultrasonic irradiation and application in sonocatalytic degradation of organic dyes. Ultrason. Sonochem..

[cit41] Li D., Li R., Zeng F., Yan W., Deng M., Cai S. (2023). The photoexcited electron transfer and photocatalytic mechanism of g-C3N4/TiO2 heterojunctions: Time-domain ab initio analysis. Appl. Surf. Sci..

[cit42] Bhatti M. A., Shah A. A., Almani K. F., Tahira A., Chalangar S. E., dad Chandio A., Nur O., Willander M., Ibupoto Z. H. (2019). Efficient photo catalysts based on silver doped ZnO nanorods for the photo degradation of methyl orange. Ceram. Int..

[cit43] Amrute V., Supin K. K., Chanda A. (2024). Observation of excellent photocatalytic and antibacterial activity of Ag doped ZnO nanoparticles. RSC Adv..

[cit44] Wagh S. S., Kadam V. S., V Jagtap C., Salunkhe D. B., Patil R. S., Pathan H. M., Patole S. P. (2023). Comparative Studies on Synthesis, Characterization and Photocatalytic Activity of Ag Doped ZnO Nanoparticles. ACS Omega.

[cit45] Imboon T., Somyanonthanakun W., Photiwut P., Khumphon J., Ghosh S., Tanna A. R., Sridawong L., Chotikaprakhan S., Thongmee S. (2025). Synthesis of Ag-doped ZnO nanoparticles for the photocatalytic degradation of phenol and dyes. E3S Web Conf..

[cit46] Ali I. O., Nady H., Mohamed M. I., Salama T. M. (2024). Fabrication and characterization of ZnO and Ag/ZnO nanoparticles for efficient degradation of crystal violet dye in aqueous solution. J. Indian Chem. Soc..

[cit47] Babele P. K., Thakre P. K., Kumawat R., Tomar R. S. (2018). Zinc oxide nanoparticles induce toxicity by affecting cell wall integrity pathway, mitochondrial function and lipid homeostasis in Saccharomyces cerevisiae. Chemosphere.

[cit48] Xu Q., He C., Xiao C., Chen X. (2016). Reactive Oxygen Species (ROS) Responsive Polymers for Biomedical Applications. Macromol. Biosci..

[cit49] Jung W. K., Koo H. C., Kim K. W., Shin S., Kim S. H., Park Y. H. (2008). Antibacterial Activity and Mechanism of Action of the Silver Ion in *Staphylococcus aureus* and *Escherichia coli*. Appl. Environ. Microbiol..

[cit50] Li Y., Liao C., Tjong S. C. (2020). Recent Advances in Zinc Oxide Nanostructures with Antimicrobial Activities. Int. J. Mol. Sci..

